# Association of a Composite Inflammatory Score with Stroke Prevalence: A Cross-Sectional Study

**DOI:** 10.3390/life16050785

**Published:** 2026-05-08

**Authors:** Boyuan Li, Yiu-Wing Kam

**Affiliations:** Division of Natural and Applied Sciences, Duke Kunshan University, No. 8 Duke Avenue, Kunshan 215316, China; bl310@duke.edu

**Keywords:** inflammatory score, stroke prevalence, C-reactive protein, white blood cell count, NHANES, cross-sectional study, systemic inflammation, risk stratification

## Abstract

Systemic inflammation plays a key role in cerebrovascular disease. While C-reactive protein (CRP) and white blood cell (WBC) count are individual risk markers, the predictive value of a combined inflammatory score (IS) for stroke prevalence remains unclear. This study aimed to investigate this association in a national population. Data were obtained from the National Health and Nutrition Examination Survey (NHANES) from 1999 to 2010. An IS was calculated as the sum of standardized Z-scores for CRP and WBC. Weighted multivariable logistic regression assessed the IS-stroke association, adjusting for demographics and clinical confounders. Restricted cubic spline (RCS) models and subgroup analyses were performed. Among 9963 included participants, 345 reported a history of stroke. After full adjustment, individuals in the highest IS quartile had 1.60-fold higher odds of stroke (OR = 1.60, 95% CI: 1.08–2.36) compared to the lowest quartile. Each unit increase in IS was associated with 6% higher odds (OR = 1.06, 95% CI: 1.01–1.12). RCS analysis revealed that higher composite IS is independently associated with increased stroke prevalence, suggesting a nonlinear association. The composite IS remained a significant predictor in models where the individual CRP and WBC components did not. This combined index may capture inflammatory burden more comprehensively than either marker alone in cross-sectional analyses. However, given the cross-sectional design, these findings should be interpreted cautiously and require confirmation in prospective studies.

## 1. Introduction

Stroke remains a primary cause of death and long-term disability worldwide. Forecasts show that the global burden of stroke will rise sharply in the coming decades. Stroke is currently the second leading cause of death and the third leading cause of combined death and disability (DALYs) globally [1]. According to the 2024 Lancet Neurology Commission, it is predicted that stroke-related deaths will increase by 50% from 2020 to 2050, growing from 6.6 million to 9.7 million. Disability-adjusted life years (DALYs) will also increase from 144.8 million to 189.3 million in this period [2]. Prevention has mostly targeted common risk factors such as hypertension, diabetes, and lifestyle [3,4]. However, new evidence shows that chronic, low-level systemic inflammation is also important in stroke development and outcomes [5,6,7].

To date, most stroke-related inflammation studies have focused on single biomarkers or specialized cohorts, leaving a gap in population-level evidence [8]. Notably, recent research has used sophisticated multi-marker models, such as the Framingham Offspring Study’s five-biomarker panel [9], showing a dose-response association between inflammation and incident stroke. However, fewer studies have evaluated whether simplified, clinically accessible composite inflammatory markers are associated with prevalent stroke at the population level.

Two widely available biomarkers (C-reactive protein (CRP) and white blood cell count (WBC)) have been consistently linked to stroke and vascular events. For example, CRP (a sensitive acute-phase reactant) is elevated in many stroke patients and has been shown in meta-analyses to predict ischemic stroke risk [10,11,12]. Similarly, elevated WBC count, an integrative marker of systemic inflammation, has been associated with increased stroke severity and mortality [13,14]. Together, CRP and WBC capture complementary aspects of the inflammatory response, and both are routinely measured in clinical practice.

Because individual inflammatory biomarkers may be influenced by multiple physiological and pathological factors, composite indices based on routine inflammatory markers have increasingly been explored in epidemiological research. One such inflammatory score (IS), calculated from the standardized (Z-score) values of CRP and WBC, has previously been associated with all-cause mortality, cardiovascular mortality [15,16], and cardiovascular disease in NHANES and other cohorts [17,18]. However, its relationship with prevalent stroke has been less clearly characterized in a nationally representative population.

Therefore, the aim of the present study was not to develop a new inflammatory index, but to evaluate whether this simple and clinically accessible composite marker is associated with prevalent stroke in U.S. adults using NHANES 1999–2010 data. In addition, we explored whether this association followed a nonlinear pattern using restricted cubic spline analysis. By focusing on a stroke-specific outcome in a nationally representative sample, this study was intended to provide population-level evidence regarding the potential epidemiological relevance of a simplified inflammatory burden indicator.

## 2. Methods

### 2.1. Study Population and Data Source

**Figure 1:**
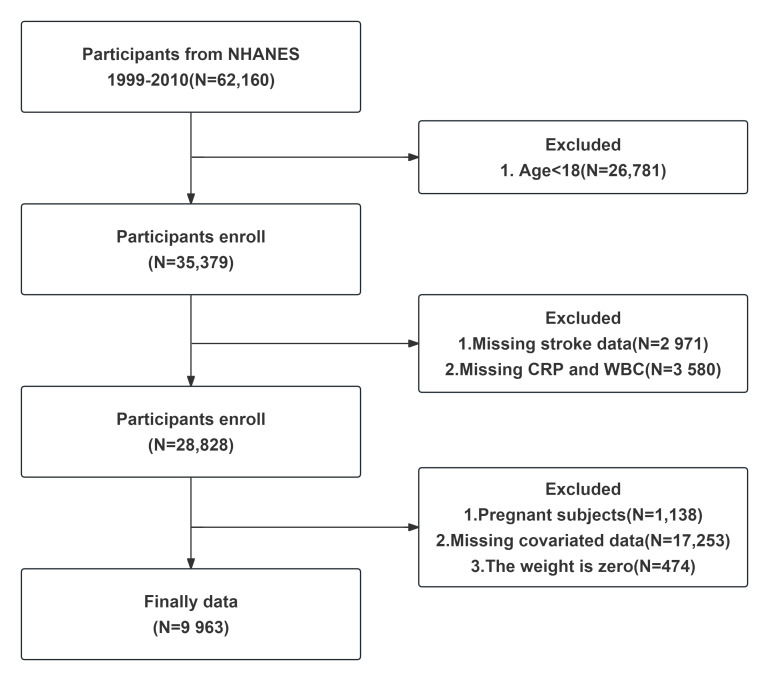
Flow chart of the sample selection from NHANES 1999–2010. Excluding cases with missing key IS related data or missing covariate data, 9963 patient data were ultimately retained for a cross-sectional study on the association between inflammation and stroke.

We used data from NHANES cycles 1999–2010 (six 2-year cycles). NHANES uses a multistage probability design with sampling weights to ensure national representativeness [19]. Participants completed interviews and examinations, including laboratory tests. We included adults (age ≥ 20) with complete data on CRP, WBC, stroke status, and covariates. Missing data were not imputed. Participants with missing values in variables required for a given descriptive analysis or regression model were excluded from that specific analysis (complete-case analysis). A flowchart of participant recruitment is shown in Figure 1. We excluded individuals with missing CRP or WBC, missing stroke questionnaire, or missing covariates (e.g., blood pressure, lipids).

Given the cross-sectional design of this study, the observed associations cannot be used to infer causality.

### 2.2. Assessment of Stroke

Stroke status in the NHANES dataset was determined from questionnaire-based self-report collected during structured interviews. Participants were classified as having a history of stroke if they answered “Yes” to the question: “Has a doctor or other health professional ever told you that you had a stroke?” Thus, the outcome represented self-reported physician diagnosis rather than adjudicated clinical records. Because detailed information on stroke subtype, timing, and imaging confirmation was unavailable, misclassification could not be excluded.

### 2.3. Variable Definitions

The inflammatory score (IS) was calculated as follows: for each participant, CRP and WBC values were standardized to z-scores (Z = (X − mean)/SD) using the study sample mean and SD, and then summed: IS = Z_CRP + Z_WBC [15,16,17]. Higher IS reflects greater systemic inflammation.

CRP was measured in mg/dL and WBC in 10^9^/L in the NHANES laboratory. Participants were grouped into quartiles (Q1–Q4) of IS. Stroke status was determined by questionnaire: participants were asked if a doctor or health professional had ever told them that they had a stroke. A positive response was classified as a prevalent stroke. Demographic covariates included age, sex, race/ethnicity, poverty-income ratio, education, and marital status. Health-related covariates included body mass index (BMI), smoking and alcohol use (current vs. not), hypertension (yes/no), diabetes (yes/no), coronary heart disease history, and other laboratory measures (blood urea nitrogen, ALT, LDL cholesterol, etc.). Definitions (e.g., for hypertension and diabetes) followed the standard NHANES criteria. All variables were drawn from NHANES interview/exam data.

### 2.4. Statistical Analysis

All analyses accounted for the NHANES complex sampling design and weights, as recommended by NHANES guidelines [20]. Descriptive statistics were weighted to the US population; categorical variables are reported as weighted % (*n*), and continuous variables as medians (interquartile range). Differences across IS quartiles were tested using a design-adjusted chi-square test for categorical variables and a Kruskal–Wallis test for medians. We used multivariable logistic regression (survey-weighted) to examine the association of IS with stroke prevalence, and prior to regression analysis, collinearity among the major continuous covariates was assessed by calculating variance inflation factors (VIFs). First, IS was entered as a continuous predictor. Then the IS quartiles were modeled (Q1 as referent). In each analysis, we constructed three models: Model 1 unadjusted; Model 2 adjusted for age, sex, and BMI; and Model 3 adjusted for age, sex, BMI, race/ethnicity, education, marital status, poverty income ratio, diabetes, hypertension, coronary heart disease, smoking, drinking, blood urea nitrogen, ALT, and LDL cholesterol. We report odds ratios (ORs) with 95% confidence intervals (CIs) and *p*-values. In addition, we ran an analysis comparing Q4 with the combined Q1–Q3 to test the effect of extreme IS elevation. Restricted cubic spline (RCS) regression was used to explore the dose-response relationship between continuous IS and stroke risk. To allow for potential nonlinearity, we compared models with 3 to 5 knots placed at default percentiles of the IS distribution. The RCS curve and *p*-values for overall and non-linear trends were examined. Restricted cubic spline regression models were used to explore the dose–response association between the IS and stroke risk. We compared alternative knot specifications and selected the 3-knot model which had the lowest Akaike Information Criterion (AIC) and was therefore selected as the primary model. Subgroup analyses tested whether the IS–stroke association differed by strata of age (<60 vs. ≥60), sex, BMI (>25 vs. ≤25), diabetes, hypertension, CHD, smoking, and alcohol use, using interaction terms. Results were summarized in a forest plot. Finally, we evaluated predictive performance by ROC analysis. We plotted weighted ROC curves for logistic models: unadjusted (Model 1), partially adjusted (Model 2), and fully adjusted (Model 3). The area under the curve (AUC) was computed and compared between models using DeLong’s test. Statistical significance was set at α = 0.05. All statistical analyses were performed using R software (version 4.2.2). *p*-values < 0.05 were considered statistically significant. Microsoft Excel (Microsoft ® Excel ® Suitable for Microsoft 365MSO (version 2604 Build 16.0.19929.20106) 64 bit) was used for data visualization.

## 3. Results

### 3.1. Baseline Characteristics

A total of 9963 participants were included and categorized into quartiles (Q1–Q4) based on IS, as detailed in Table 1. The median age was 45 years, and 51% were female. Compared with those in the lowest quartile (Q1), participants in the highest quartile (Q4) were more likely to be female (57% vs. 50%), current smokers (61% vs. 37%), and have higher BMI (median 30 vs. 25), lower educational attainment, and higher prevalence of diabetes (19% vs. 8.3%) and hypertension (41% vs. 29%) (all *p* < 0.001). In terms of laboratory markers, CRP, WBC, TG, HbA1c, and fasting glucose levels were significantly elevated in Q4 compared to Q1, while HDL-C levels were lower (all *p* < 0.001). These results suggest that a higher inflammatory burden, as reflected by IS, is associated with more adverse metabolic and vascular risk profiles.

IS values were significantly different between participants with and without stroke, as shown in Supplementary Figure S1. The median IS was higher in people with stroke than in those without. A Welch’s *t*-test showed a significant difference between the groups (*p* < 0.001), indicating a clear difference in systemic inflammation by stroke status.

### 3.2. Associations Between IS, WBC, CRP, and Stroke

Collinearity diagnostics for IS and the major continuous metabolic covariates are shown in Supplementary Figure S2, indicating no evidence of severe collinearity among the primary continuous covariates (all VIFs < 10).

Table 2 shows the weighted logistic regression results. As a continuous predictor, IS was significantly associated with stroke: in unadjusted analyses, each 1-unit increase in IS corresponded to a 12% higher odds of stroke (OR 1.12, 95% CI: 1.07–1.18, *p* < 0.001). After adjusting for age, sex, and BMI (Model 2), OR = 1.09 (1.03–1.14, *p* = 0.002). In the fully adjusted model (Model 3), IS remained a significant predictor (OR 1.06, 95% CI: 1.00–1.13, *p* = 0.046). When comparing IS quartiles, the highest quartile (Q4) had substantially greater stroke odds than Q1. In Model 3, Q4 vs. Q1 gave OR 1.61 (95% CI: 1.01–2.57, *p* = 0.045). Models 1, 2 showed even stronger effects. By contrast, WBC and CRP individually showed attenuated associations after full adjustment. For example, WBC Q4 vs. Q1 had OR 1.35 (0.93–1.97, *p* = 0.12) in Model 3 (non-significant), and CRP Q4 vs. Q1 had OR 1.61 (0.99–2.62, *p* = 0.054). The *p* for trend across IS quartiles was significant in unadjusted and partially adjusted models, but marginal in the full model.

To further evaluate the impact of elevated inflammatory burden on stroke, we compared individuals in the highest quartile of the inflammatory score (Q4) with those in the combined lower three quartiles (Q1–Q3) (Table 3). In the unadjusted model (Model 1), participants in Q4 had significantly higher odds of stroke compared to those in Q1–Q3 (OR = 1.79, 95% CI: 1.37–2.33, *p* < 0.001). This association remained robust after adjusting for age, gender, and BMI (Model 2: OR = 1.61, 95% CI: 1.01–1.82, *p* = 0.002). In the fully adjusted model (Model 3), which accounted for sociodemographic factors, comorbidities, and laboratory parameters, the association remained statistically significant (OR = 1.36, 95% CI: 1.20–2.15, *p* = 0.044). These findings indicate that individuals in the highest quartile of IS, reflecting elevated systemic inflammation, exhibit a significantly increased risk of stroke independent of conventional vascular risk factors.

### 3.3. Predictive Performance of Inflammatory Score Models

**Figure 2:**
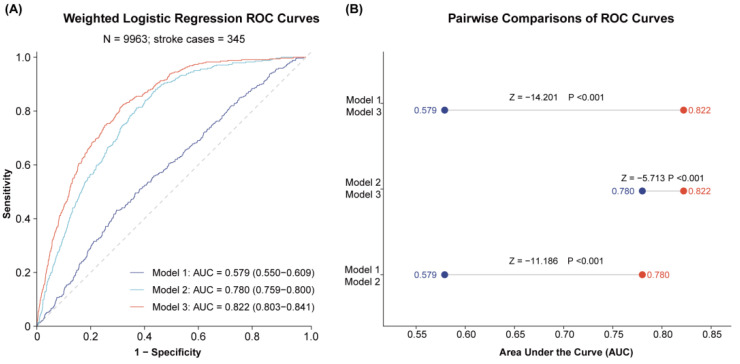
Weighted logistic regression ROC curves for stroke prediction in Models 1–3 (as described in Methods). (**A**) ROC curves for Model 1 (unadjusted, IS alone), Model 2 (adjusted for age, sex, and BMI), and Model 3 (fully adjusted for sociodemographic, behavioral, and clinical covariates). (**B**) Pairwise comparisons of AUC values between the three models. Model 1 had an AUC of 0.579, Model 2 had an AUC of 0.780, and Model 3 had an AUC of 0.822. The increasing AUC values indicate improved overall discriminative performance of progressively adjusted models; however, this pattern should not be interpreted as the isolated predictive contribution of IS alone. The grey dotted diagonal line represents the performance of a random classifier (AUC = 0.5).

The overall discriminative performance of progressively adjusted logistic regression models for prevalent stroke was evaluated using weighted logistic regression models, and the results are presented as ROC curves in Figure 2.

Model 1, which was unadjusted, showed poor predictive performance with an AUC of 0.579. After adjusting for age, gender, and BMI in Model 2, the AUC increased markedly to 0.780. Further adjustment has been made in Model 3 to include a comprehensive set of covariates, including race, education, marital status, PIR, diabetes, hypertension, coronary heart disease, smoking, drinking, BUN, ALT, and LDL-C, resulting in the highest discriminative capacity (AUC = 0.822).

Pairwise comparisons of AUCs were statistically significant (all *p* < 0.001). These findings indicate that the overall discriminative performance improved with progressive covariate adjustment.

### 3.4. Dose-Response Relationship Between IS and Stroke

**Figure 3:**
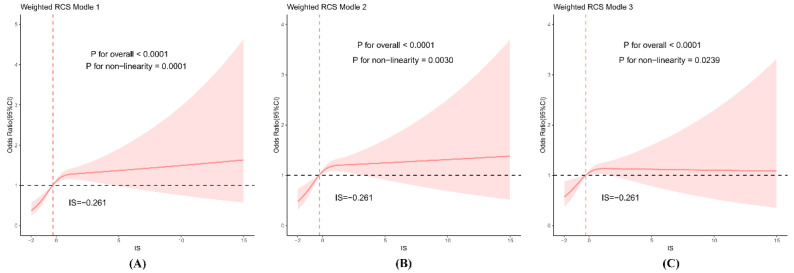
Dose–response relationship between inflammatory score (IS) and odds of stroke using restricted cubic spline regression in weighted models. (**A**) Unadjusted model (Model 1); (**B**) Model 2 adjusted for age, gender, and BMI; (**C**) Model 3 adjusted for age, gender, BMI, race/ethnicity, education, marital status, poverty-income ratio, diabetes, hypertension, coronary heart disease, smoking, drinking, blood urea nitrogen, ALT, and LDL-C.

Figure 3 demonstrated restricted cubic spline regression models used to explore the dose–response association between the IS and stroke risk. In the unadjusted model (Model 1), a significant nonlinear relationship was observed (*p* for non-linearity = 0.0001; *p* for overall association <0.0001). The odds of stroke remained stable at low IS levels but increased steeply once IS exceeded the reference point (IS = −0.261).

In Model 2, adjusted for age, gender, and BMI, the nonlinear pattern persisted (*p* for non-linearity = 0.0030; *p* for overall <0.0001), with stroke risk rising progressively across increasing IS values. After further adjustment for socioeconomic, behavioral, and clinical covariates (Model 3), the association between IS and stroke remained statistically significant (*p* for overall <0.0001), and the non-linearity remained detectable (*p* = 0.0239), although slightly attenuated.

Across all models, the dose-response curves suggested a nonlinear association between IS and prevalent stroke. Odds remained relatively stable at lower IS levels and became more clearly elevated at higher IS levels. However, the detailed shape of the curve, particularly at the extremes, should be interpreted cautiously because it may be influenced by knot specification and sparse observations.

### 3.5. Subgroup Analysis

**Figure 4:**
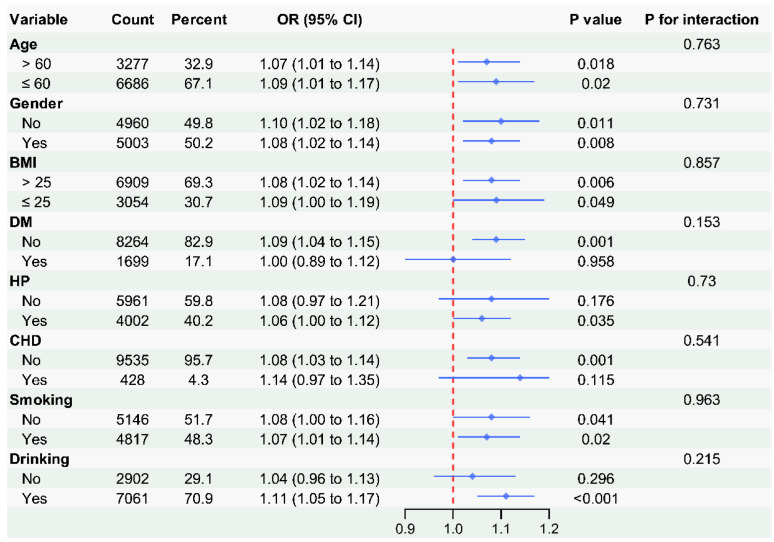
Subgroup analysis forest plot: odds of stroke per 1-unit increase in inflammatory score, by subgroup. The analysis reveals positive associations (OR > 1.00 per 1-unit IS increase) in all subgroups, formal interaction testing confirmed no significant effect modification by any demographic or clinical variable (all *p*-interaction > 0.05), indicating a generally consistent direction of association across subgroups, although the subgroup analyses should be interpreted cautiously given the limited number of stroke cases and the absence of statistically significant interaction terms.

To assess the stability of the association between the IS and stroke, stratified analyses were performed across several population subgroups (Figure 4). Overall, the positive relationship between IS and stroke was consistent across most strata.

In both younger participants (aged 60 years or below) and older participants (aged 60 years or above), elevated IS was associated with increased odds of stroke, with odds ratios of 1.09 (95% CI: 1.01–1.17) and 1.07 (95% CI: 1.01–1.14), respectively. No significant interaction was observed between age and IS (*p* for interaction = 0.763). Similarly, the association between IS and stroke was comparable in males and females, with odds ratios of 1.10 and 1.08, respectively, and no significant interaction (*p* = 0.731). Stratification by body mass index (BMI > 25 kg/m^2^ vs. ≤25 kg/m^2^) yielded similar results, with no interaction (*p* = 0.857).

Among individuals without diabetes, IS was significantly associated with stroke risk (OR = 1.09, 95% CI: 1.04–1.15), while the association was not statistically significant in those with diabetes (OR = 1.00, 95% CI: 0.89–1.12). However, the interaction between IS and diabetes status did not reach statistical significance (*p* = 0.153). Consistent patterns were observed in subgroups defined by hypertension (*p* for interaction = 0.730), coronary heart disease (*p* = 0.541), smoking status (*p* = 0.963), and alcohol consumption (*p* = 0.215).

Importantly, in all subgroups, the point estimates for IS remained above 1.00, suggesting a uniform direction of effect. The absence of statistically significant interaction effects suggests that the direction of association was generally similar across subgroups; however, these subgroup analyses should be interpreted cautiously given the limited number of stroke cases.

## 4. Discussion

In this nationally representative analysis, a higher composite IS was independently associated with greater odds of stroke. Even after adjusting for traditional risk factors, each unit increase in IS was associated with a higher stroke risk (fully adjusted OR ~1.06 per unit). Subjects in the top IS quartile had about 1.6 times the odds of stroke as those in lower quartiles. These results suggest that the combined burden of inflammation, as captured by CRP and WBC, is associated with prevalent stroke at the population level. In the fully adjusted models, the composite IS remained statistically associated with prevalent stroke, whereas the individual CRP and WBC components did not retain statistical significance. This finding suggests that the composite score may provide a pragmatic summary of inflammatory burden in this dataset.

Recent stroke-specific evidence further supports an inflammatory contribution [21,22,23,24]. Meta-analyses have shown that each unit rise in high-sensitivity CRP is associated with a 27% increase in the risk of poor functional outcome or recurrence after stroke [25]. Likewise, prospective cohorts demonstrate that higher total or neutrophil WBC counts predict incident and fatal stroke, independent of conventional risk factors [13,26]. Our findings extend these single-marker observations by showing that a simple composite retains significance after multivariable adjustment, whereas the individual markers do not. The spline analysis suggested a nonlinear association, with the odds of prevalent stroke increasing more clearly at higher IS levels. Mechanistically, pronounced elevation of CRP/WBC likely demonstrates amplified innate immune activation, endothelial dysfunction, and pro-thrombotic states that promote cerebrovascular events. Some prior studies have suggested that very low CRP levels may be associated with frailty or altered physiological status in certain populations, including higher all-cause mortality [21,22,26]. However, the present cross-sectional analysis does not allow us to infer that this mechanism explains the lower-tail pattern of the spline curve.

From a clinical perspective, the implications of IS should be interpreted cautiously. Although CRP and WBC are routinely measured in clinical settings, the present cross-sectional study does not support the use of IS as a screening tool or as a basis for therapeutic decision-making. At this stage, IS may be more appropriately viewed as a simple epidemiological indicator of inflammatory burden at the population level [13,26]. Whether it has incremental value for screening, risk stratification, or clinical management will require confirmation in prospective longitudinal studies. Also, although the fully adjusted model containing the IS showed the highest discriminative capacity (AUC = 0.822), it is critical to note that this improved AUC reflects the contribution of the entire multivariate model, including many established risk factors, and should not be interpreted as the isolated predictive contribution of the IS itself.

Nonetheless, several limitations should be acknowledged. First, the cross-sectional design of NHANES precludes any inference regarding temporality or causality. Because stroke status in this study reflected a self-reported history of prior stroke, whereas CRP and WBC were measured at the time of the survey examination, the stroke event may have occurred months or even years before biomarker assessment. As a result, the temporal sequence between inflammatory burden and stroke cannot be established. Reverse causation therefore cannot be ruled out, and elevated inflammatory markers may partly reflect persistent physiological changes, comorbidity burden, or residual inflammatory consequences after stroke rather than antecedent exposure contributing to stroke development. Second, stroke history was self-reported and may be subject to recall bias or misclassification, and information on stroke subtypes was unavailable. Third, although we adjusted for a broad range of demographic and clinical variables, residual confounding cannot be excluded. Some potentially relevant factors, such as medication use (e.g., statins or anti-inflammatory drugs), prior or concurrent infections, and other transient inflammatory conditions, were not consistently available or could not be comprehensively accounted for in the present analysis. In addition, CRP and WBC are non-specific markers that may be influenced by multiple acute and chronic processes. Therefore, a single cross-sectional measurement may not fully represent the long-term inflammatory burden relevant to stroke.

## 5. Conclusions

In conclusion, a higher inflammatory score, reflecting combined CRP and WBC elevation, was associated with prevalent stroke in U.S. adults. These findings support a cross-sectional link between systemic inflammatory burden and stroke history, but they do not establish temporality or causality. Prospective longitudinal studies are required to determine whether this composite score has predictive value for incident stroke and whether it may have future clinical utility.

## Figures and Tables

**Table 1:** Baseline characteristics of study participants, stratified by inflammatory score quartiles (IS Q1–Q4).

Characteristic	Overall (*n* = 9963)	Q1 (*n* = 2386)	Q2 (*n* = 2473)	Q3 (*n* = 2555)	Q4 (*n* = 2549)	*p* Value
**Demographic characteristics**						
Age, years	45 (33, 58)	45 (34, 57)	45 (33, 59)	46 (33, 59)	45 (33, 58)	>0.9
Sex, *n* (%)						<0.001
Female	4960 (51%)	1142 (50%)	1154 (48%)	1236 (50%)	1428 (57%)	
Male	5003 (49%)	1244 (50%)	1319 (52%)	1319 (50%)	1121 (43%)	
**Race/ethnicity,** ***n*** **(%)**						<0.001
Mexican American	1942 (7.2%)	376 (5.8%)	482 (7.0%)	583 (8.6%)	501 (7.5%)	
Non-Hispanic White	5221 (73%)	1193 (72%)	1331 (75%)	1327 (73%)	1370 (73%)	
Non-Hispanic Black	1827 (10%)	590 (13%)	420 (9.5%)	366 (8.2%)	451 (10%)	
Other race	973 (9.2%)	227 (9.1%)	240 (8.8%)	279 (10%)	227 (8.6%)	
**Marital status,** ***n*** **(%)**						<0.001
Never married	1504 (16%)	383 (16%)	395 (17%)	369 (16%)	357 (16%)	
Married/living with partner	6274 (66%)	1594 (71%)	1567 (67%)	1634 (66%)	1479 (61%)	
Divorced/separated/widowed	2185 (18%)	409 (13%)	511 (16%)	552 (18%)	713 (23%)	
**Education,** ***n*** **(%)**						<0.001
<High school	2783 (18%)	533 (13%)	647 (16%)	795 (21%)	808 (22%)	
=High school	2360 (25%)	493 (21%)	582 (24%)	619 (26%)	666 (30%)	
>High school	4820 (57%)	1360 (66%)	1244 (60%)	1141 (53%)	1075 (48%)	
**PIR,** ***n*** **(%)**						<0.001
≤1	1749 (12%)	326 (9.1%)	399 (11%)	447 (12%)	577 (17%)	
1–3	4190 (36%)	874 (30%)	1023 (35%)	1145 (39%)	1148 (40%)	
>3	4024 (52%)	1186 (61%)	1051 (54%)	963 (49%)	824 (43%)	
**Lifestyle and clinical characteristics**						
BMI, kg/m^2^	27 (24, 32)	25 (23, 29)	27 (24, 30)	28 (24, 32)	30 (26, 36)	<0.001
**Smoking,** ***n*** **(%)**						<0.001
No	5146 (51%)	1490 (63%)	1346 (55%)	1262 (48%)	1048 (39%)	
Yes	4817 (49%)	896 (37%)	1127 (45%)	1293 (52%)	1501 (61%)	
**Drinking,** ***n*** **(%)**						0.13
No	2902 (25%)	696 (24%)	675 (24%)	751 (25%)	780 (27%)	
Yes	7061 (75%)	1690 (76%)	1798 (76%)	1804 (75%)	1769 (73%)	
**Diabetes,** ***n*** **(%)**						<0.001
No	8264 (87%)	2109 (92%)	2103 (89%)	2100 (87%)	1952 (81%)	
Yes	1699 (13%)	277 (8.3%)	370 (11%)	455 (13%)	597 (19%)	
**Hypertension,** ***n*** **(%)**						<0.001
No	5961 (66%)	1581 (71%)	1526 (69%)	1473 (64%)	1381 (59%)	
Yes	4002 (34%)	805 (29%)	947 (31%)	1082 (36%)	1168 (41%)	
**CHD,** ***n*** **(%)**						0.068
No	9535 (97%)	2303 (97%)	2363 (96%)	2449 (97%)	2420 (96%)	
Yes	428 (3.4%)	83 (2.6%)	110 (3.6%)	106 (3.4%)	129 (4.2%)	
**Study-related variables**						
**Stroke,** ***n*** **(%)**						<0.001
No	9618 (97%)	2334 (98%)	2391 (97%)	2467 (98%)	2426 (96%)	
Yes	345 (2.5%)	52 (1.5%)	82 (2.5%)	88 (2.4%)	123 (3.7%)	
IS	−0.33 (−0.90, 0.43)	−1.21 (−1.43, −1.04)	−0.62 (−0.76, −0.47)	0.01 (−0.16, 0.21)	1.16 (0.74, 1.93)	<0.001
CRP, mg/dL	0.19 (0.07, 0.43)	0.07 (0.04, 0.15)	0.14 (0.07, 0.26)	0.24 (0.11, 0.43)	0.66 (0.29, 1.31)	<0.001
WBC, ×10^9^/L	6.40 (5.40, 7.80)	4.80 (4.30, 5.20)	6.10 (5.70, 6.40)	7.20 (6.80, 7.70)	8.90 (7.90, 10.00)	<0.001

Data are presented as unweighted counts (weighted %) for categorical variables and weighted median (Q1, Q3) for continuous variables. Extended laboratory variables are provided in Supplementary Table S1.

**Table 2:** Weighted Logistic Regression Analysis of Stroke and IS/WBC/CRP.

Variables	Model 1	Model 2	Model 3
**IS continuous**	1.12 (1.07–1.18) <0.001	1.09 (1.03–1.14) 0.002	1.06 (1.00–1.13) 0.046
Q1	REF	REF	REF
Q2	1.70 (1.08–2.65) 0.021	1.47 (0.94–2.31) 0.090	1.36 (0.86–2.15) 0.2
Q3	1.58 (0.98–2.55) 0.062	1.33 (0.83–2.14) 0.2	1.16 (0.72–1.87) 0.5
Q4	2.54 (1.63–3.97) <0.001	2.07 (1.31–3.28) 0.002	1.61 (1.01–2.57) 0.045
*p* for trend	<0.001	0.004	0.075
**WBC continuous**	1.06 (1.02–1.11) 0.005	1.04 (1.01–1.08) 0.012	1.04 (1.00–1.07) 0.033
Q1	REF	REF	REF
Q2	1.31 (0.89–1.95) 0.2	1.21 (0.81–1.80) 0.3	1.14 (0.76,1.72) 0.5
Q3	1.17 (0.82–1.67) 0.4	1.06 (0.75–1.50) 0.7	0.98 (0.69,1.40) 0.9
Q4	1.76 (1.24–2.50) 0.002	1.71 (1.19–2.45) 0.004	1.35 (0.93–1.97) 0.12
*p* for trend	0.004	0.009	0.2
**CRP continuous**	1.16 (1.08–1.24) <0.001	1.10 (1.01–1.20) 0.032	1.07 (0.97–1.17) 0.2
Q1	REF	REF	REF
Q2	2.02 (1.19–3.43) 0.009	1.37 (1.81–2.33) 0.2	1.29 (0.76–2.19) 0.3
Q3	2.30 (1.30–4.07) 0.005	1.35 (0.78–2.36) 0.2	1.17 (0.68–2.02) 0.6
Q4	3.54 (2.21–5.66) <0.001	1.98 (1.21–3.24) 0.007	1.61 (0.99–2.62) 0.054
*p* for trend	<0.001	0.010	0.080

**Table 3:** Weighted Logistic Regression Analysis of Stroke and IS.

IS	Q1–Q3	Q4		
		OR	95% CI	*p* Value
Model 1	REF	1.79	1.37–2.33	<0.001
Model 2	REF	1.61	1.01–1.82	0.002
Model 3	REF	1.36	1.20–2.15	0.044

Model 1: unadjusted. Model 2: adjusted for Age, Gender and BMI. Model 3: adjusted for Age, Gender, BMI, Race, Education, Marital status, PIR, DM, HP, CHD, Smoking, Drinking, BUN, ALT, LDL-C.

## Data Availability

The original contributions presented in this study are included in the article. Further inquiries can be directed to the corresponding author. All data used in this study are publicly available through the NHANES website (https://www.cdc.gov/nchs/nhanes/ (accessed on 25 June 2025)).

## Supplementary Materials

The following supporting information can be downloaded at: https://www.mdpi.com/article/10.3390/life16050785/s1. Supplementary Figure S1. Box plot of inflammatory score distribution by stroke status. Stroke cases show significantly higher median and wider spread of IS than non-stroke cases. A heteroskedasticity-adjusted Welch’s *t*-test was conducted to assess group differences. Statistical significance is exhibited as *** *p* < 0.001. Supplementary Figure S2. Collinearity diagnostics for IS and metabolic covariates. (A) Pairwise correlation heatmap among IS and selected metabolic variables. (B) Variance inflation factor (VIF) values for the corresponding continuous covariates. These results did not indicate severe multicollinearity. Supplementary Table S1. Extended laboratory characteristics according to inflammatory score quartiles.
