# Supplementary material for: Association of a Composite Inflammatory Score with Stroke Prevalence: A Cross-Sectional Study
Source: Life (Basel). 2026 May 8;16(5):785. doi: 10.3390/life16050785 (PMC13208536; doi:10.3390/life16050785)
Supplement: Supplementary file 1 [file life-16-00785-s001.zip › Supplementary Figure S1.pdf]

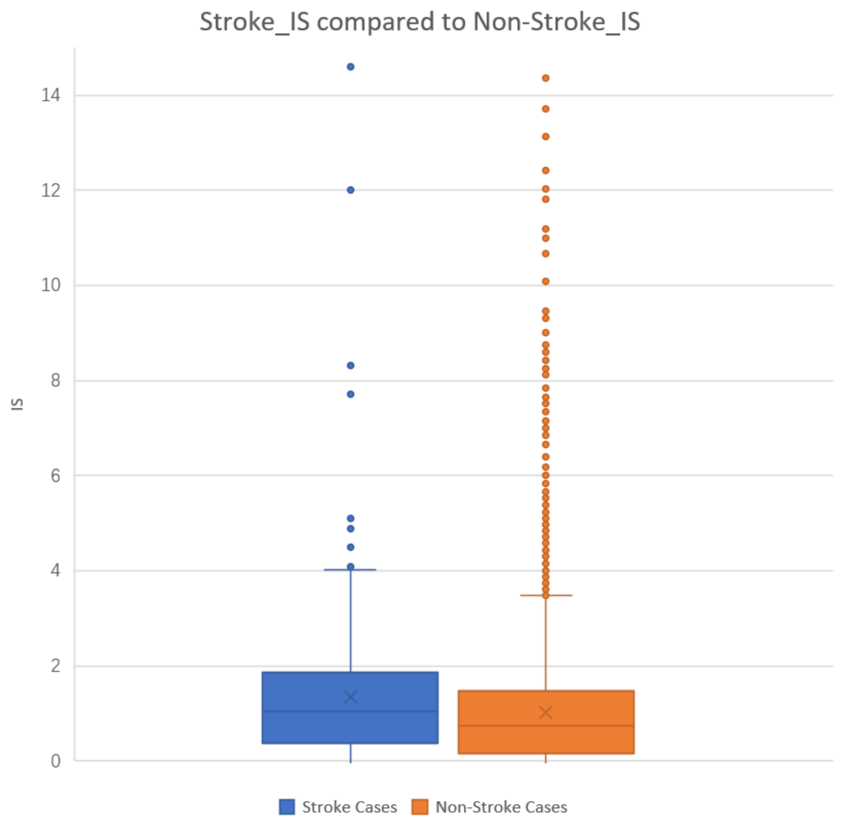

Supplementary Figure S1. Box plot of inflammatory score distribution by stroke status. Stroke cases show significantly higher median and wider spread of IS than non-stroke cases. A heteroskedasticity-adjusted Welch's t-test was conducted to assess group differences. Statistical significance is exhibited as ***p<0.001.
