# Supplementary material for: Association of a Composite Inflammatory Score with Stroke Prevalence: A Cross-Sectional Study
Source: Life (Basel). 2026 May 8;16(5):785. doi: 10.3390/life16050785 (PMC13208536; doi:10.3390/life16050785)
Supplement: Supplementary file 1 [file life-16-00785-s001.zip › Supplementary Figure S2.pdf]

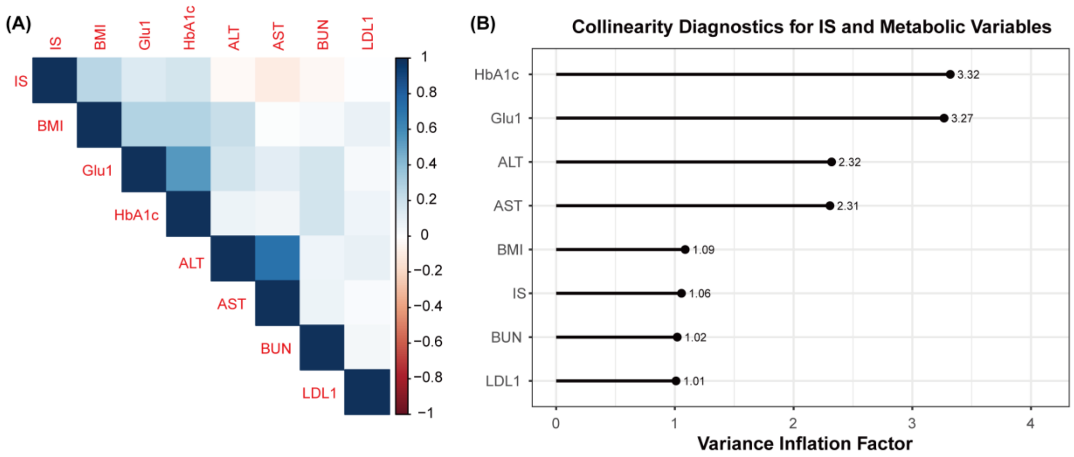

Supplementary Figure S2. Collinearity diagnostics for IS and metabolic covariates. (A) Pairwise correlation heatmap among IS and selected metabolic variables. (B) Variance inflation factor (VIF) values for the corresponding continuous covariates. These results did not indicate severe multicollinearity.
