# Supplementary material for: Association of a Composite Inflammatory Score with Stroke Prevalence: A Cross-Sectional Study
Source: Life (Basel). 2026 May 8;16(5):785. doi: 10.3390/life16050785 (PMC13208536; doi:10.3390/life16050785)
Supplement: Supplementary file 1 [file life-16-00785-s001.zip › Supplementary Table S1.pdf]

Supplementary Table S1

Extended laboratory characteristics according to inflammatory score quartiles

Characteristic	Overall (n = 9,963)	Q1 (n = 2,386)	Q2 (n = 2,473)	Q3 (n = 2,555)	Q4 (n = 2,549)	P value
FBG, mmol/L	5.38 (5.03, 5.83)	5.29 (4.97, 5.66)	5.38 (5.00, 5.80)	5.44 (5.05, 5.93)	5.50 (5.11, 6.05)	<0.001
HbA1c, %	5.30 (5.10, 5.60)	5.30 (5.00, 5.50)	5.30 (5.10, 5.60)	5.40 (5.10, 5.70)	5.40 (5.20, 5.80)	<0.001
ALT, U/L	21 (17, 29)	21 (17, 28)	22 (17, 29)	22 (17, 30)	21 (16, 29)	0.002
AST, U/L	23 (19, 27)	23 (20, 27)	23 (20, 27)	23 (19, 27)	22 (18, 27)	<0.001
BUN, mmol/L	4.30 (3.57, 5.40)	4.64 (3.57, 5.71)	4.64 (3.57, 5.71)	4.30 (3.57, 5.40)	4.28 (3.21, 5.36)	<0.001
TG, mmol/L	1.25 (0.88, 1.78)	1.02 (0.73, 1.41)	1.23 (0.87, 1.73)	1.35 (0.96, 1.90)	1.46 (1.04, 2.06)	<0.001
TC, mmol/L	5.02 (4.40, 5.74)	5.04 (4.37, 5.74)	5.02 (4.40, 5.72)	5.04 (4.45, 5.77)	4.99 (4.32, 5.74)	0.5
LDL-C, mmol/L	3.00 (2.43, 3.60)	3.00 (2.41, 3.57)	3.00 (2.41, 3.60)	3.03 (2.48, 3.65)	2.97 (2.41, 3.62)	0.12
HDL-C, mmol/L	1.32 (1.09, 1.60)	1.45 (1.22, 1.78)	1.34 (1.14, 1.60)	1.27 (1.06, 1.55)	1.22 (1.03, 1.50)	<0.001

Note. Data are presented as unweighted counts (weighted %) for categorical variables and weighted median (Q1, Q3) for continuous variables.
